# Myxoma of the kidney – an unusual benign renal tumor: a case report

**DOI:** 10.1186/s13256-016-1194-y

**Published:** 2017-02-14

**Authors:** Somuah Tenkorang, Youssef Kharbach, Jean-Paul Omana, Boubacar Efared, Soufiane Mellas, Mohammed Fadl Tazi, Mohamed Sekal, Taoufik Harmouch, Abdelhak Khallouk, Jamal Mohammed El Fassi, Jalal Eddine El Ammari, Moulay Hassan Farih

**Affiliations:** 1 Department of Urology, Hassan II Teaching Hospital, Fez, Morocco; 2 Department of Anatomo-pathology, Hassan II Teaching Hospital, Fez, Morocco

**Keywords:** Renal myxoma, Benign tumor, CT scan, Nephrectomy

## Abstract

**Background:**

Myxomas are rare benign soft tissue tumors. The kidney is an unusual location for this tumor. For this reason, less than 15 cases of renal myxoma have been reported in the English literature. There are no specific clinical and radiological features reported for this tumor that allow a preoperative diagnosis enabling a conservative treatment.

**Case presentation:**

We report another case of renal myxoma found in a 50-year-old Moroccan woman who presented with a right dull flank pain. An abdominal computed tomography scan objectified a suspected malignant renal mass. Thus, radical nephrectomy was performed. Histopathology of the specimen revealed the typical appearance of a myxoma.

**Conclusions:**

The objective of this report is to add another case report of this rare benign renal tumor to the literature. This benign tumor with excellent prognosis has no specific preoperative features that could justify a conservative management; a radical approach remains the therapeutic option for now.

## Background

Myxomas are rare benign soft tissue tumors. Renal myxomas are rare; very few cases have been reported in the English literature. There are no specific clinical and radiological features reported for this tumor that allow a preoperative diagnosis enabling a conservative treatment. We report a case of renal myxoma diagnosed after radical nephrectomy had been performed for a suspected malignant renal tumor. We present a computed tomography (CT) scan and the histopathological findings of the case.

## Case presentation

A 50-year-old Moroccan woman presented to our hospital for assessment of a right dull flank pain that had begun a year ago. She had a medical history of hypertension and type 2 diabetes mellitus and was under calcium channel blocker and antidiabetic medication respectively.

**Fig. 1:**
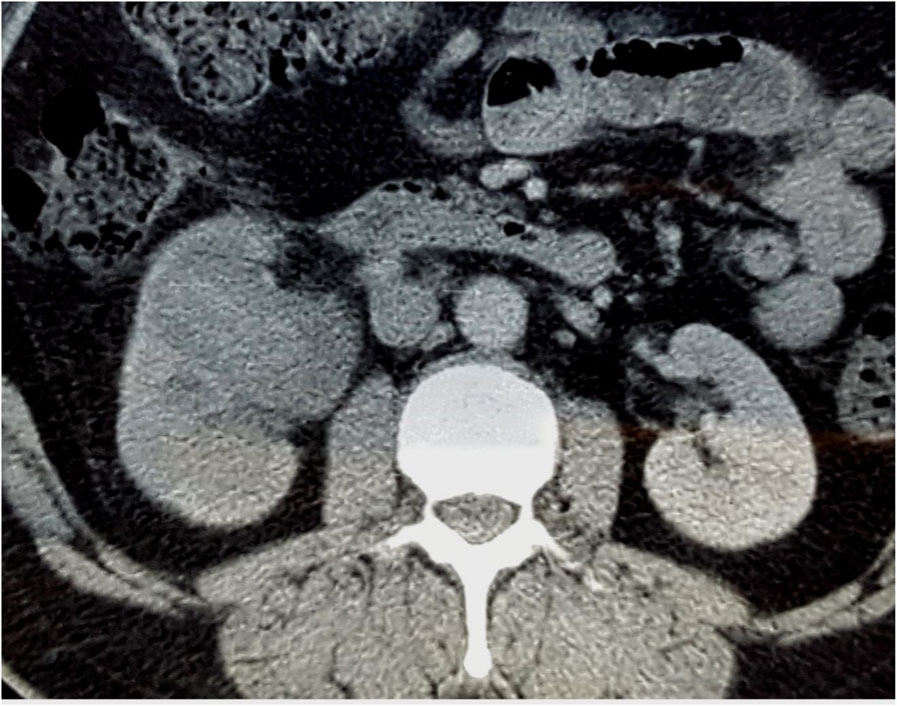
Contrast-enhanced computed tomography scan showing a slightly enhanced mass of the mid-portion of the kidney

A physical examination found no palpable mass in her abdomen but a slight right flank pain. All laboratory investigations were within normal limits. An abdominal CT scan objectified a hypodense well-defined mass in the mid-portion of her right kidney. It measured 4 × 3.5 cm and was slightly enhanced after intravenous contrast measuring 61 Hounsfield unit (HU; Fig. 1).

**Fig. 2:**
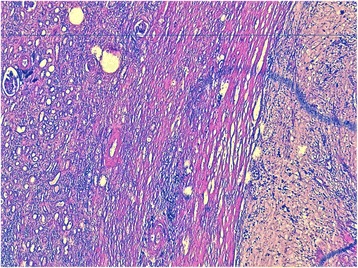
A well-demarcated tumor. Fewer cells, with abundant myxoid stroma, separated from the normal renal parenchyma (*left*; hematoxylin, erythrosine, and saffron × 50).

**Fig. 3:**
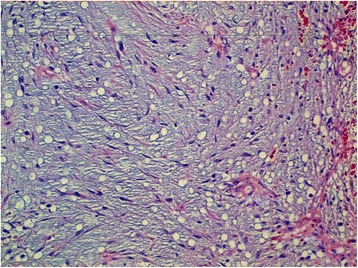
Spindle cells dispersed in an abundant myxoid stroma. Hematoxylin, erythrosine, and saffron × 200
- The myxoid stroma focally and slightly stained positive with S100 protein, epithelial membrane antigen (EMA), and Ki-67 but stained negatively with pancytokeratin and CD 34 (Fig. 4).

She underwent radical nephrectomy for a suspected malignant renal tumor. The specimen was sent for histopathological examination:

- A gross description of her right kidney measured 16 × 9 × 6 cm. A cut section showed a solid cystic gelatinous mass in the mid-portion of her kidney.
- Microscopic examination revealed spindle cells with a hypervascular myxoid stroma with areas of hypercellularity. Moderate atypical cells with no mitosis were observed. No capsular invasion was found. Adjacent renal parenchyma had areas of chronic pyelonephritis (Figs. 2, 3).

**Fig. 4:**
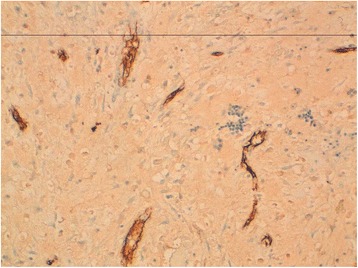
Tumor cells do not express CD34 (×100). Very few vessels in the stroma stained positive with CD 34

Thus, the final diagnosis of renal myxoma was made. She is well and asymptomatic at 3 months follow up.

## Discussion

Myxomas are rare benign soft tissue tumors that mainly occur in the heart and skin, although various anatomical locations have been described for this tumor [1]. Renal myxomas are very rare; very few cases have been reported in the English literature. In 1887, Hulke reported “a large capsular myxoma of the kidney” [2], which was the first reported case of renal myxoma [3–6]. To the best of our knowledge, approximately 15 cases of renal myxoma have been reported since Hulke’s first reported case.

There are challenges with respect to its preoperative diagnosis as no specific clinical manifestation has been reported so far due to its rare occurrence, which limits its study. Documented cases of this tumor have shown nearly equal prevalence in males and females. Although the majority of reported cases of renal myxomas have been diagnosed incidentally, flank pain is the main clinical presentation in symptomatic patients for this tumor as in our case [7]. Ultrasound can detect a renal mass but CT scans and magnetic resonance imaging (MRI) have been promising in diagnosing and managing this disease [8]. It usually has an appearance of a cystic solid mass highly suspicious for malignancy, which is similar to our case. This finding justified radical nephrectomy for almost all the cases, which was followed by histopathological examination confirming the diagnosis of a pure renal myxoma as observed in our case. The availability of advanced imaging modalities, such as positron emission tomography (PET), CT, or CT-guided biopsy, may help confirm the benign nature of this disease and differentiate it from other benign and malignant soft tissue tumors with very similar features. This will certainly justify conservative treatments for this tumor allowing the preservation of unaffected parenchyma in the remnant kidney [3, 5, 8]. It is also encouraging to know that the prognosis of renal myxoma is excellent due to its benign nature. No case of recurrence has been reported so far [7].

## Conclusions

Myxomas of the kidney are very rare benign soft tissue tumors. Preoperative diagnosis of this disease allowing conservative treatment remains a challenge due to its very rare occurrence and its lack of specific clinical and radiological manifestations. Histopathological examination after radical nephrectomy remains the sure diagnostic tool for this disease. Advanced imaging modalities and investigations will probably help in diagnosing and managing this disease. This benign tumor has a good prognosis with no known recurrence.

## Abbreviations

CT: Computed tomography
EMA: Epithelial membrane antigen
HES: Hematoxylin, erythrosine, and saffron
HU: Hounsfield unit
PET: Positron emission tomography
